# A mathematical model of case-ascertainment bias: Applied to case-control studies nested within a randomized screening trial

**DOI:** 10.1371/journal.pone.0194608

**Published:** 2018-03-19

**Authors:** Rick J. Jansen, Bruce H. Alexander, Richard B. Hayes, Anthony B. Miller, Sholom Wacholder, Timothy R. Church

**Affiliations:** 1 Department of Public Health, North Dakota State University, Fargo, North Dakota, United States of America; 2 Division of Environmental Health Sciences, University of Minnesota School of Public Health, Minneapolis, Minnesota, United States of America; 3 Division of Epidemiology, NYU School of Medicine, New York, New York, United States of America; 4 Dalla Lana School of Public Health, University of Toronto, Toronto, Ontario, Canada; 5 Division of Cancer Epidemiology and Genetics, National Cancer Institute, Bethesda, Maryland, United States of America; Karolinska Institutet, SWEDEN

## Abstract

When some individuals are screen-detected before the beginning of the study, but otherwise would have been diagnosed symptomatically during the study, this results in different case-ascertainment probabilities among screened and unscreened participants, referred to here as lead-time-biased case-ascertainment (LTBCA). In fact, this issue can arise even in risk-factor studies nested within a randomized screening trial; even though the screening intervention is randomly allocated to trial arms, there is no randomization to potential risk-factors and uptake of screening can differ by risk-factor strata. Under the assumptions that neither screening nor the risk factor affects underlying incidence and no other forms of bias operate, we simulate and compare the underlying cumulative incidence and that observed in the study due to LTBCA. The example used will be constructed from the randomized Prostate, Lung, Colorectal, and Ovarian cancer screening trial. The derived mathematical model is applied to simulating two nested studies to evaluate the potential for screening bias in observational lung cancer studies. Because of differential screening under plausible assumptions about preclinical incidence and duration, the simulations presented here show that LTBCA due to chest x-ray screening can significantly increase the estimated risk of lung cancer due to smoking by 1% and 50%. Traditional adjustment methods cannot account for this bias, as the influence screening has on observational study estimates involves events outside of the study observation window (enrollment and follow-up) that change eligibility for potential participants, thus biasing case ascertainment.

## Introduction

Previous literature describes how the screening process itself can bias observational studies designed to estimate the potential benefit of following a specific screening protocol in a population (i.e., screening efficacy studies)[1]. The possible effect screening may have on observational studies of other risk factors has only been mentioned in the context of modifying mortality risk and decreasing incidence through removal of precursor lesions[2,3]. A previous model attempted to quantify this indirect effect screening use has on ascertainment[4] independent of these other effects. Our group has modified this model to estimate the impact of ascertainment bias on risk factor studies of cancer [5]. Traditional adjustment methods cannot account for this bias, as the influence screening has on observational study estimates involves events outside of the study observation (enrollment and follow-up) window causing changes in eligibility for potential participants, thus biasing case ascertainment. Adjustment based on simply observing and measuring screening in the population and adjusting for this variable through stratification or in a regression model cannot properly account for this bias.

The theoretical, unobservable interval between the time a case is screen-detected and the time he/she would have been diagnosed symptomatically in the absence of screening is called lead-time. The lead-time interval represents the length of time that diagnosis of disease has been advanced because of the use of screening. When this lead-time interval overlaps either the beginning or end of the case ascertainment period (e.g., a person is screen-detected before the beginning of the study, but otherwise would have been diagnosed systematically during the study), this can lead to different case-ascertainment probabilities among screened and unscreened participants, referred to as lead-time-biased case-ascertainment (LTBCA)[4]. Because exposures are not randomized in observational risk-factor studies of cancer and may be correlated with screening uptake both before and during the study, different strata of the risk factor can exhibit different screening patterns. This can lead to different degrees of LTBCA, and thereby change the observed cumulative incidence differently between strata, biasing the corresponding relative risk estimate.

In this article, we modify a previously described mathematical model[4,5] to yield a more realistic estimate for the potential amount of LTBCA bias in a risk-factor study and provide R[6] statistical programing [R Foundation for Statistical Computing, Vienna, Austria] code (S1 File) so other researchers can run and adapt the model. Under the assumptions that neither screening nor the risk factor affects underlying incidence or mortality and no other forms of bias operate, we simulate and compare the underlying cumulative incidence and that expected in the study due to LTBCA; these estimates can be used as a theoretical bias correction factor. As an illustrative example, this model is applied to a case-control study nested within the Prostate, Lung, Colorectal, and Ovarian cancer (PLCO) screening trial. Because unobserved parameters cannot be known directly, a sensitivity analysis is performed to examine a range of plausible bias factors and to highlight how different model parameters affect bias estimates.

### Mathematical model

A progressive disease model[7] and counterfactual concept [8] were used to develop the mathematical model, which simulates the cumulative incidence expected in the sampled population under the factual and the counterfactual conditions regarding screening behavior. A previous model to assess LTBCA on estimates of screening efficacy has been described in detail elsewhere, along with its derivation[5]. We will focus on the model modifications made to improve it below with more detail presented in a supporting document to this article (S2 File).

The formulae relating the level of screening use in a population to the case selection for a specific study (i.e. observed cumulative disease incidence during a study) has been given previously[5]. Here *w*(*x*) is the preclinical incidence function, *f*(*z—x*) is the preclinical duration density function, where *z* is time of symptomatic diagnosis and *x* is time of detectable, preclinical disease onset. Modified from Jansen et al.[5] and presented below, *a* is a specified time, *a*_*E*_ is end of the study period, *a*_*0*_ is the beginning of the study period, *i* represents the number of 5-year age categories within our study population, **ω** = <ω_i_> is age structure of the study population, *k*_*1*,*r*_(*x*) is the age, risk-factor dependent screening proportion function, and *k*_*2*,*r*_*(x)* is the age, risk-factor dependent screening rate function. Functions before and during the enrollment period are represented with a “_b” and “_d” respectively, and were added to the above symbols for the preclinical incidence, screening proportion, and screening rate functions. These functions change over calendar year and so creating a before and during function averaged over calendar years for each of the respective periods is done to capture some of this variability while limiting the complexity of the model.

The mathematical formula used within each age and risk-factor stratum when the outcome of interest is **incidence** follows:

Assuming in the target population nobody is ever screened, the cumulative incidence (*G*_*U-R*_*(·)*) for risk-factor stratum *r* and age stratum *i* to age *a* is:

$$G_{U-R}(a_{E,i};r)=\int_{0}^{a_{0,i}}w\_b(x)\int_{a_{0,i}}^{a_{E,i}}f(z-x)\;dzdx+\int_{a_{0,i}}^{a_{E,i}}w\_d(x)\int_{a_{0,i}}^{a_{E,i}}f(z-x)\;dzdx$$

The notation “;r” on the left hand side of the equation above is used to indicate that the formula is applied for each risk factor stratum (e.g. among the smoking group and non-smoking group). For a given age category, *a*_*i*_, within a given risk-factor stratum, *G*_*U-R*_(*a*_*i*_;*r*) represents the cumulative incidence rate we expect to see during the study for that specific age within our population where detectable preclinical disease onset must occur at some age, *a*, between birth (0) and age at end of the study period (*a*_*E*_) and symptomatic diagnosis must occur at some age, *a*, during the study period (between age at enrollment, *a*_*0*_, and age at end of study period, *a*_*E*_). In the model, the preclinical incidence function before the study, *w_b*(·), will only contribute incidence rates up to age at the beginning of the case-ascertainment period, *a*_*0*_, and the preclinical incidence function for during the study, *w_d*(·), will only contribute incidence rates for ages during the case-ascertainment period, *a*_*E*_. Setting *a*_*i*_ = *a*_*E*,*i*_ yields the true cumulative incidence through the end of the study for age stratum *i*.

The cumulative incidence rate for risk-factor stratum *r*, *G*_*U-R*_(*r*), is the sum over all age categories represented by our sampled population, *i* = 1,…*I*, applying the age structure of the sampled population, *ω*,_*i*_, as weights.

$$\text{GU-R}(\text{r})=\sum_{i}\omega_{i}G_{U-R}(a_{i};r)$$

Assuming the target population has the same screening behavior as the sampled population (additional incidence added to the study because of screening) and there are no other forms of bias present:

$$\begin{array}{ll} G_{S-R}(a_{E,i};r) & =\int_{0}^{a_{0,i}}w\_b(x)\left[k\_b_{1,r}(x){\left(1-\xi \right)}^{\int_{\text{min}(a_{0,i},screenage\ldots )}^{a_{0,i}}k\_b_{2,r}(y)dy}[k\_d_{1,r}(x)\left[1-{\left(1-\xi \right)}^{\int_{a_{0,i}}^{a_{E,i}}k\_d_{2,r}(y)dy}\right]\right]\int_{a_{E,i}}^{\text{max}age}f(z-x)\;dzdx\\ & +\int_{a_{0,i}}^{a_{E,i}}w\_d(x)\left[k\_d_{1,r}(x)\left[1-{\left(1-\xi \right)}^{\int_{screenage}^{a_{E,i}}k\_d_{2,r}(y)dy}\right]\right]\int_{a_{E,i}}^{\text{max}age}f(z-x)dzdx\\ & -\int_{0}^{a_{0,i}}w\_b(x)\left[k\_b_{1,r}(x)\left[1-{\left(1-\xi \right)}^{\int_{screenage}^{a_{0,i}}k\_b_{2,r}(y)dy}\right]\right]\int_{a_{0,i}}^{a_{E,i}}f(z-x)dzdx \end{array}$$

The same preclinical incidence and preclinical duration functions are used as in the model for the target population under no screening. So for a given age, *a*, *G*_*S-R*_*(·)* represents the cumulative incidence rate expected after incorporating, a fixed screening sensitivity (ξ), the proportion screened, *k_b*_*1*_(·) and the screening rate among those who screen, *k_b*_*2*_(·), before the study period and the proportion screened, *k_d*_*1*_(·) and the screening rate among those who screen, *k_d*_*2*_(·), during the study period. This model has three parts. The first line identifies the additional cumulative incidence for the members of the target population who have preclinical disease before the study starts, undergo screening starting at the age the population is recommended to start screening, represented by variable *screenage*, and are screen-detected during the ascertainment period, but, in the absence of screening, would be symptomatically diagnosed after the end of the study ascertainment period. The second line represents the cumulative incidence expected among a similar group of individuals, but their preclinical disease onset is during the case-ascertainment period and they are screen detected at during that period, but had they not been screened would have been symptomatically diagnosed after the study period. Both the first and second lines quantify the cumulative incidence added to a study because of the use of screening in the target population. The last line represents the cumulative incidence eliminated from the study due to the subjects whose preclinical disease onset and screen-detection is before the beginning of the case-ascertainment period, but had they not been screened would have been symptomatically diagnosed during the ascertainment period. The adjusted cumulative incidence rate for age stratum *i*, *G*_*S-R*_(*r*), is summed over all age strata *i = 1*,*…*, *I*, applying the age structure of the sampled population, *ω*_*i*_, as weights (same as when we assume population is unscreened).

$$\text{GS-R}(\text{r})=\sum_{i}\omega_{i}G_{S-R}(a_{i};r)$$

The ratio of these two cumulative incidences (G_S-R_(r = 1)/ G_S-R_(r = 2)) is 1 for an unbiased study. Any deviation from 1 represents LTBCA.

### Example

The example used will be constructed from the Prostate, Lung, Colorectal, and Ovarian (PLCO) cancer randomized trial[9] in which the screening test used for lung cancer was chest x-ray. The mathematical model discussed above will be applied to two nested studies to evaluate the potential for screening biased risk estimates in observational lung cancer studies. Plausible parameter values were selected for the model based on a combination of representative national registry and PLCO data.

### PLCO design

The PLCO study design has been described in detail elsewhere[10,11]. Briefly, a primary goal of the PLCO study was to examine the potential benefit of a specific screening protocol for four different cancers (i.e., Prostate, Lung, Colorectal, Ovarian). Enrollment for the PLCO study began in 1993 and ran through 2001 with approximately 77,000 men and 77,000 women aged 55–74 being followed for at least 13 years[12,13]. Participants were obtained from ten sites around the United States.

**Fig 1:**
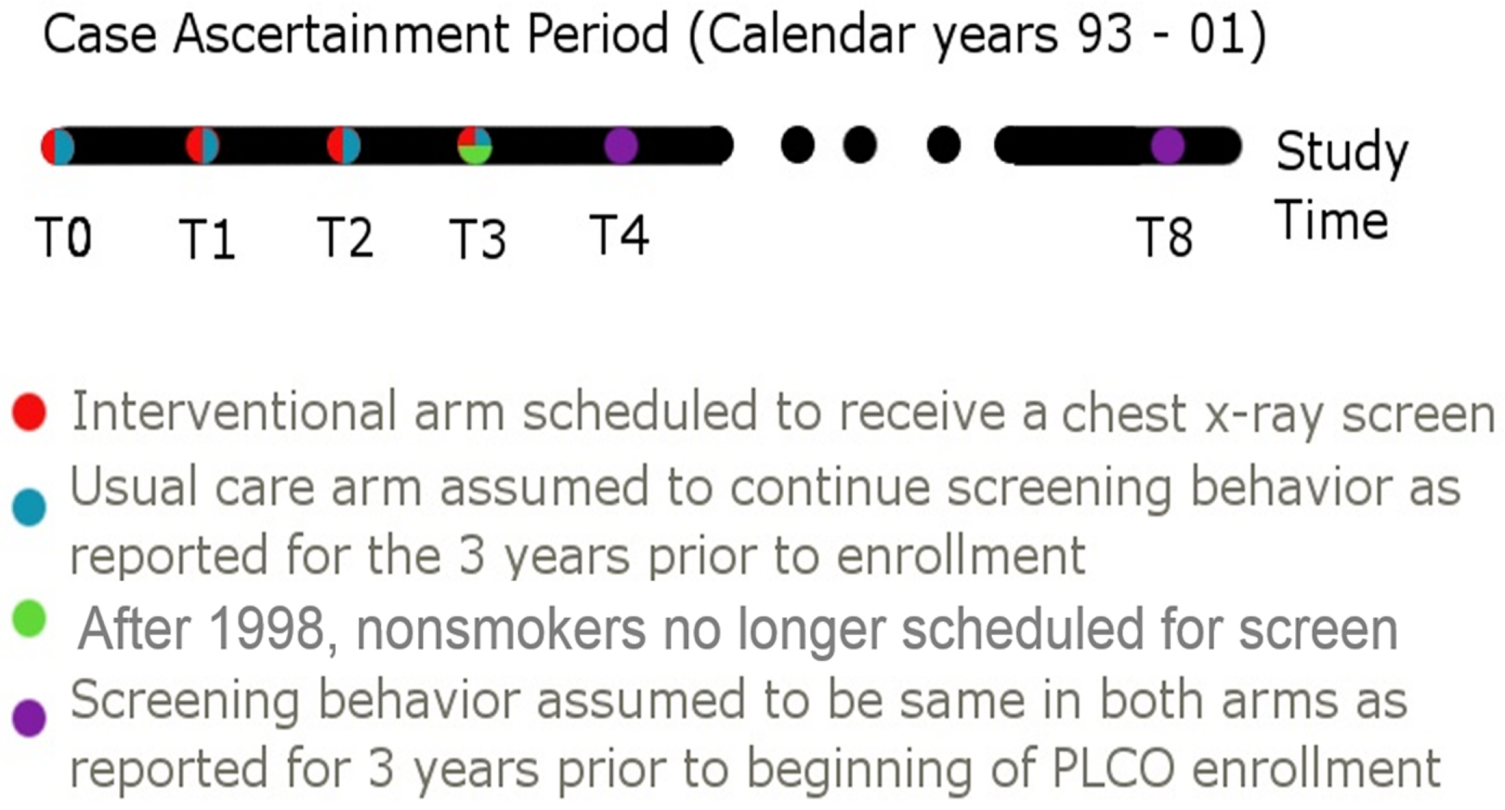
Illustration of the case-ascertainment period for the PLCO randomized trial with identification of study years and screening protocols. Individuals in the intervention arm are scheduled to receive 4 total chest x-ray screens based on the initial protocol with a 1998 modification reducing the total number screens offered to nonsmokers to 3. For the purposes of simulating screening bias here in any study year where screening information was not collected, it was assumed that individuals would continue screening behaviors as reported on the baseline questionnaire for before the beginning of the trial.

When using this example data, the focus was to determine the potential for chest x-ray screening to influence the observed relationship between smoking and lung cancer. Smoking status and screening behavior in the 3 years prior to enrollment in the PLCO study were assessed on the baseline questionnaire. The participants were randomized into an intervention or usual-care arm. The chest x-ray screening protocol for individuals in the intervention arm was to receive 3 annual screens (4 including baseline screen). However, a procedural modification in December 1998 eliminated the final reexamination for never smokers in the intervention arm[11]. This modification creates an inherently large difference in the proportion screened when comparing the ever to never smokers at study time T3 (Fig 1). This modification is integral to our research providing an identified connection between level of screening and estimated OR.

### Parameterization of the model

In order to understand the potential for screening to bias case-ascertainment in the case-control studies nested in the PLCO trial, parameters are representative of the target population–the United States. To isolate bias caused by screening, simulations incorporated a joint null hypothesis of no effect of the risk factor or chest x-ray screening on the preclinical incidence of lung cancer. Under these conditions, the true risk estimate is 1 with any systematic deviation representing bias. The statistical analysis, graphical output, and simulations were developed and run using R version 3.2.5[6].

#### Preclinical duration distribution

**Fig 2:**
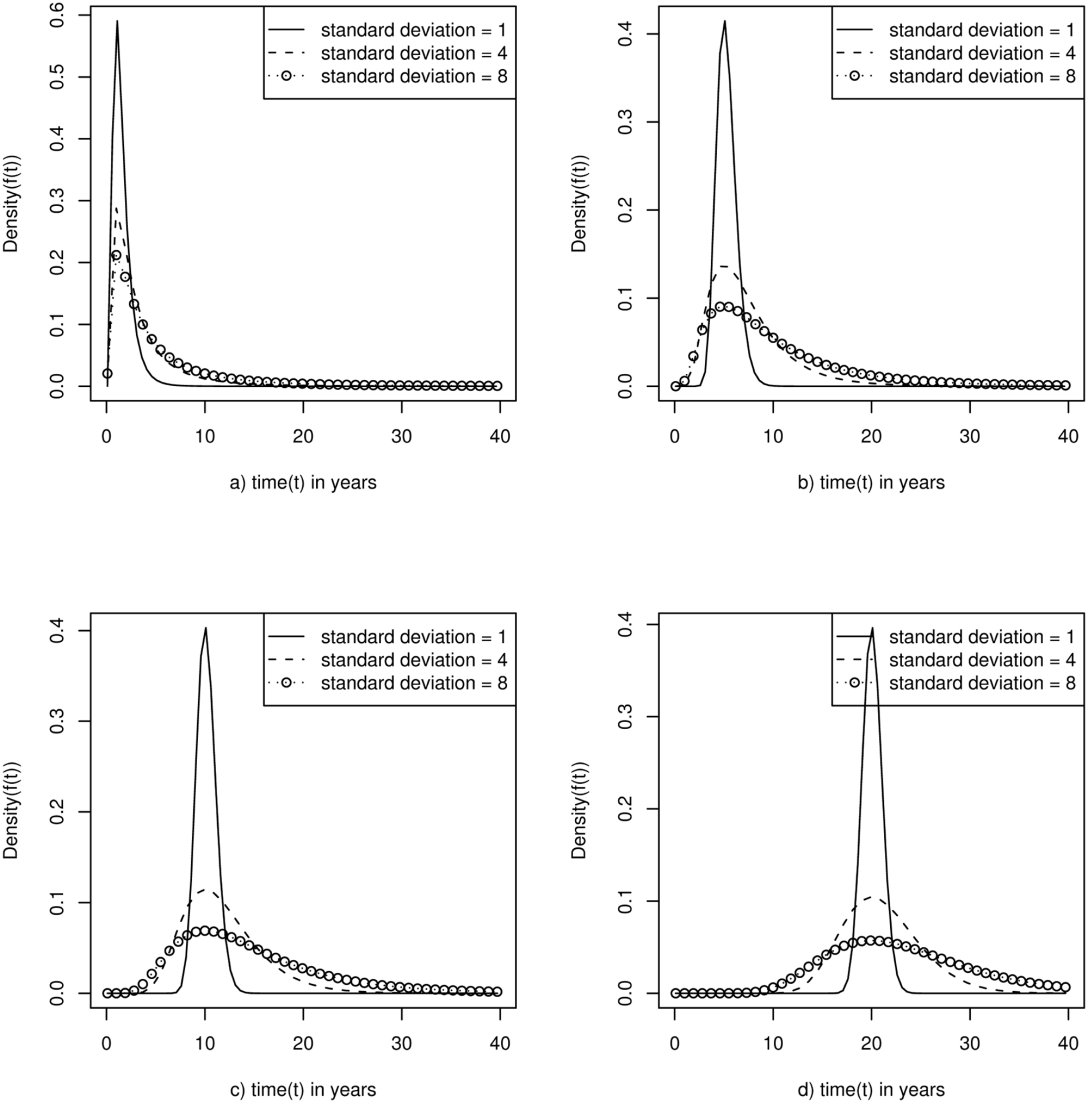
Presentation of several plausible preclinical duration distributions for lung cancer based on a log normal distribution with standard deviations of 1, 3, and 5 years for each of the following modes: 1 (a), 3 (b), 5 (c), and 10 (d). The log normal distributions are used to represent the distribution for the lengths of time individuals in our population spend in the detectable, preclinical state assuming no screening in the population. Because the preclinical duration distribution is unknown for lung cancer, the model sensitivity to variation in these parameters is explored by using the 12 different combinations. The points have no value and are just used to help distinguish between the different standard deviations within each plot.

It is known that lung cancer has different incidence rates based on different population characteristics (e.g., race, age, family history); it is also conceivable that the preclinical duration (*f*(*z—x*)) may vary based on those specific population characteristics. Because the true distribution of preclinical duration for lung cancer is unknown, a sensitivity analysis has been performed by simulating several plausible values for the mode (1, 3,5,10 years) and standard deviation (1, 3, 5 years) of the assumed lognormal distribution (Fig 2). Because our sample is mostly homogeneous with respect to race and family history and to limit complexity, an age-specific lognormal distribution is used as it meets the requirement to remain strictly positive overall values.

#### Preclinical incidence function

**Fig 3:**
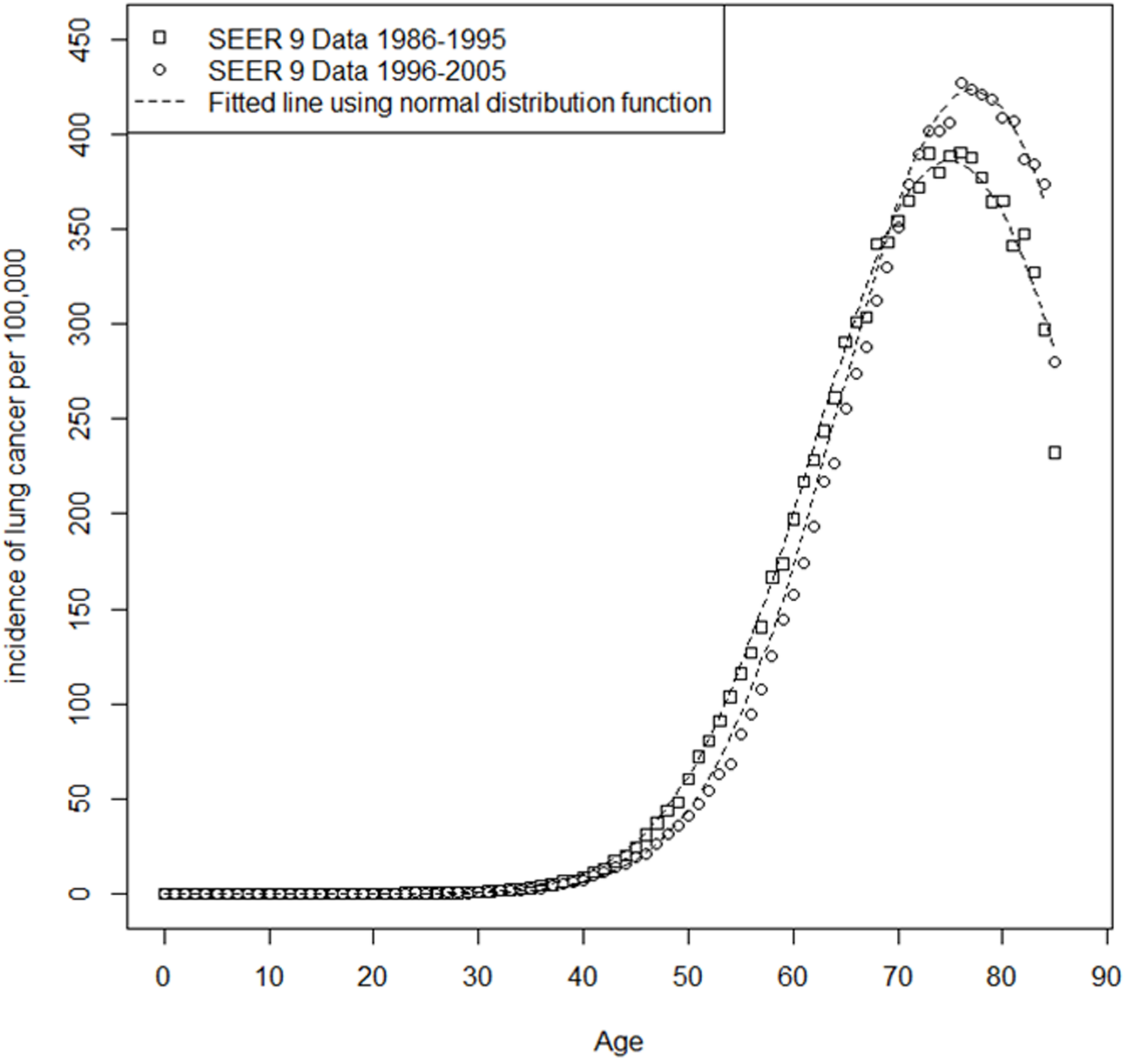
Relationship of age (age range 0–85+) to incidence rate (per 100,000) of lung cancer based on average SEER 9 registry data from 1986 to 2005. A continuous age-specific incidence intensity function was fit to the point estimates from the SEER data using non-linear minimization (dotted line). The square points identify the data points used to create the preclinical incidence function in the population before the beginning of the study and the circular points identify the data points used to create the preclinical incidence function in the population during the study. Since preclinical incidence is unobservable to get a representative preclinical incidence function the continuous incidence function represented above is shifted backward by the assumed mean of the preclinical duration distribution. For example, if the mean of the preclinical duration distribution is 5, the incidence observed for a 55 year old becomes the preclinical incidence for a 50 year old.

The SEER 9 registry[14] provides estimates for the incidence rate of lung cancer by age in the entire U.S. population based on nine long standing cancer registries. For the simulation, the focus was specifically on the years 1986–2005. Over this time period, we observed a significant shift in the incidence distribution across calendar year. To limit the level of complexity of the model while still incorporating this trend, the years 1986–1995 and 1996–2005 were used separately to get two different sets of averaged incidence points by age representing rates before and during the PLCO study, respectively. A curve was fit to each set of average age-specific lung cancer incidence rate data points from SEER9 (Fig 3) and shifted by the mean of the preclinical duration to produce an age-specific preclinical incidence distribution (*w*(*x*)) to use in our simulations. It is important to note that the incidence rate points used to derive the preclinical incidence curves are assumed to have been collected in a sample of the United States population that exhibit similar screening behaviors for smokers and nonsmokers because it is assumed here the strata have the same curve.

#### Screening intensity function

**Fig 4:**
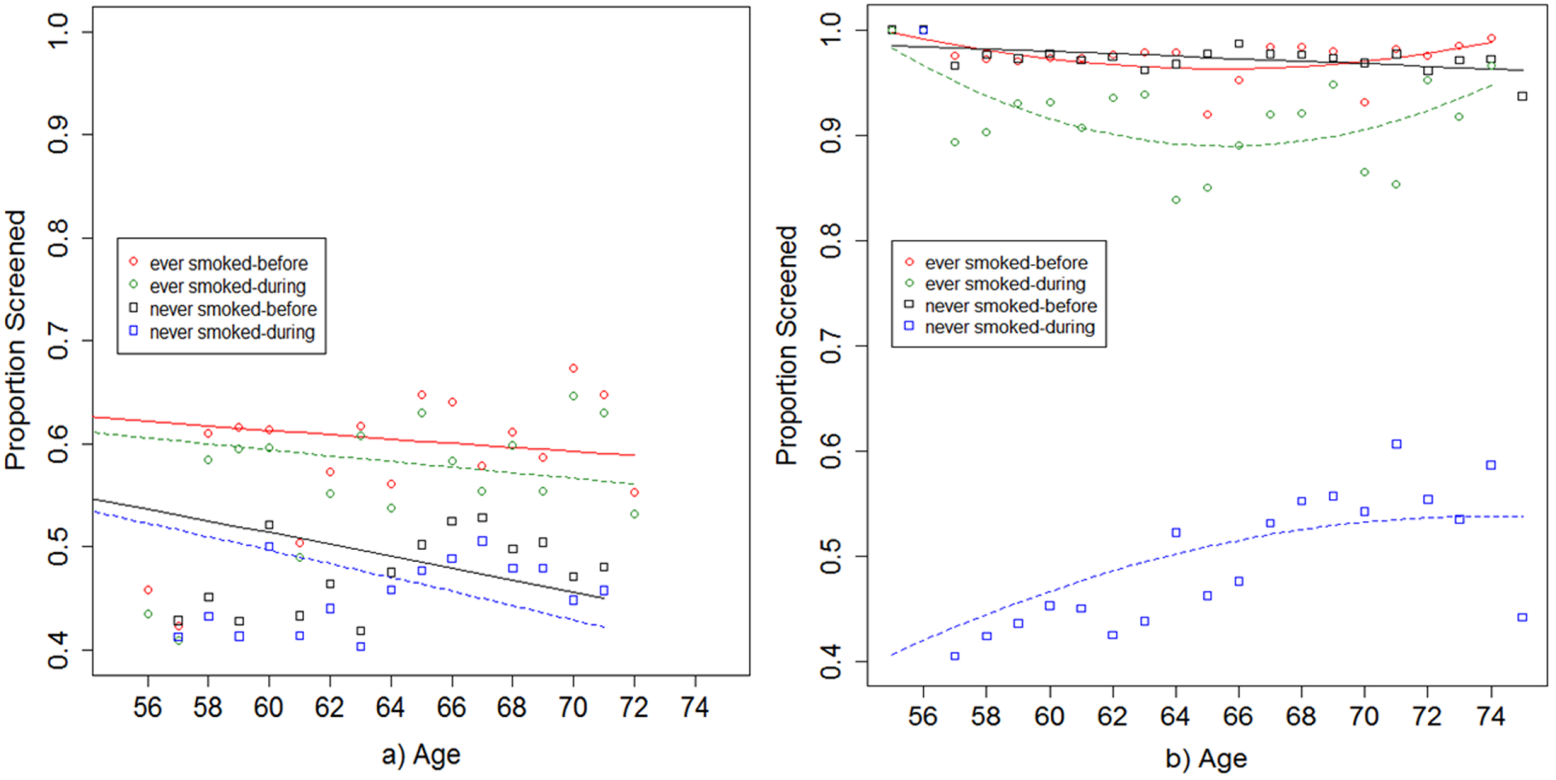
Representation of the proportion screened both before and during the study for ever smokers and never smokers separately. The age specific proportion screened (number of participants who ever received a chest x-ray test out of total number of participants at each age) is plotted for a) study sampling from all PLCO calendar enrollment years (93–01) in the usual-care arm of the PLCO during the study years T3 to T5 and b) study sampled only those affected by the procedural modifciation (95–01) in the intervention arm of the PLCO during the study years T3 to T5. Screening information from the 3 years prior to the beginning of the PLCO study and study times T0-T2 is used to calculate the proportion screened functions for before the study and screening information collected for T3 along with information from the 3 years prior to the beginning of the PLCO is used to calculate the proportion screened during the study enrollment period for each study design.

**Fig 5:**
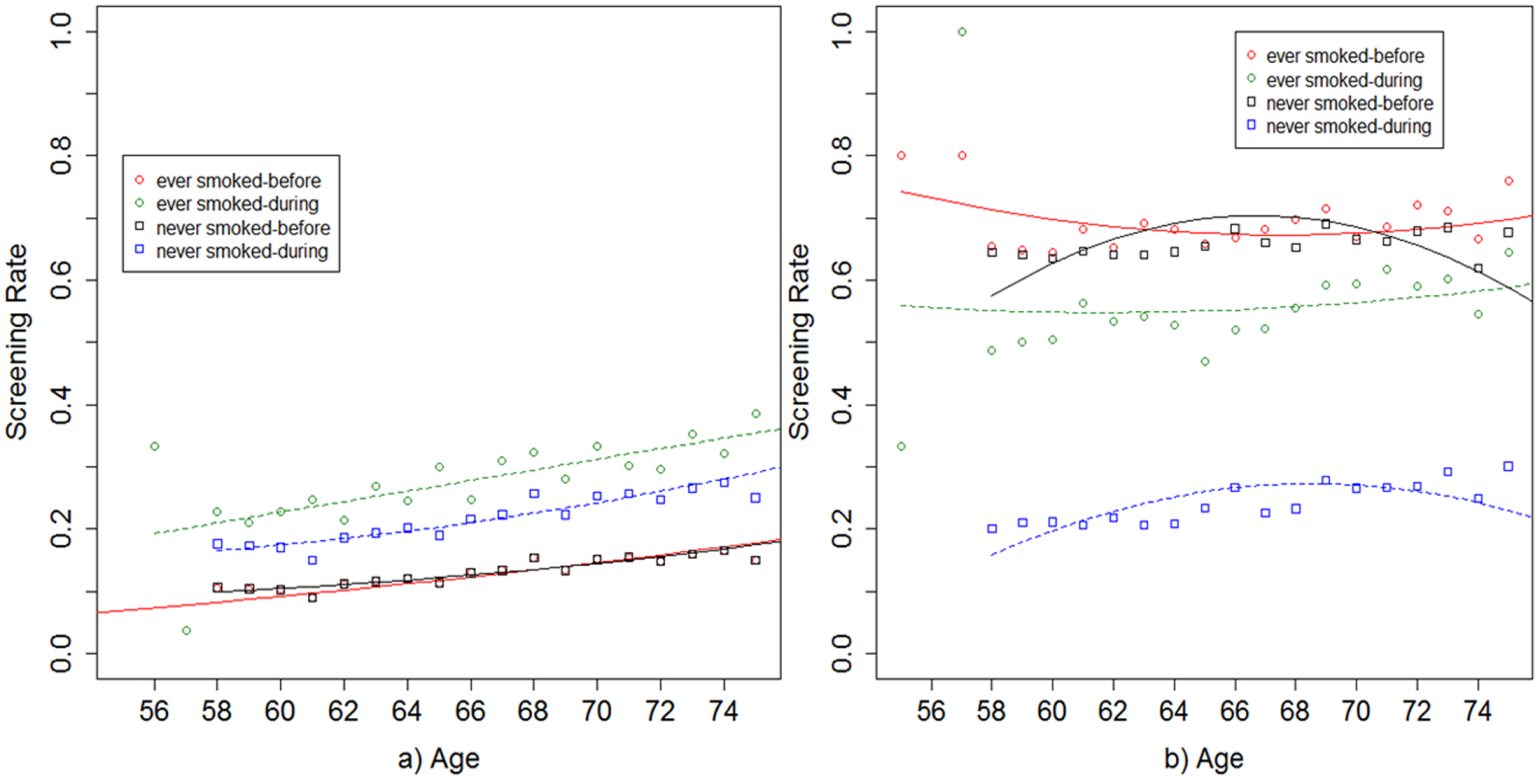
Representation of the screening rate both before and during the study for ever smokers and never smokers separately. The screening rate per year (# of screens received divided by number of years in period) among those screened is displayed for a) study sampling from all PLCO calendar enrollment years (93–01) in the usual-care arm of the PLCO during the study years T3 to T5 and b) study sampled only those affected by the procedural modification (95–01) in the intervention arm of the PLCO during the study years T3 to T5. Screening information from the 3 years prior to the beginning of the PLCO study and study times T0-T2 is used to calculate the screening rate functions for before the study and screening information collected for T3 along with information from the 3 years prior to the beginning of the PLCO is used to calculate the screening rate during the study enrollment period for each study design.

To measure screening behavior, the PLCO participants were asked if in the three years prior to their enrollment at study time T0, they ever had received a chest x-ray and the number of times they were screened (categories: 0,1,2+) during that period. Based on the baseline questionnaire, the age-specific proportion screened (*k_b*_*1*_(·) and *k_d*_*1*_(·);Fig 4) and the age-specific rate of screening (*k_b*_*2*_(·) and *k_d*_*2*_(·);Fig 5) were determined for both smokers and nonsmokers. Three different constant sensitivities of 0.46, 0.66, and 0.86 were assumed for the chest x-ray screening test to incorporate literature-based variations in these estimates and test model sensitivity to this parameter[15,16]. Generally, the only model parameter modification needed in a specific study when changing between risk factors within the same study is the age-stratum-specific equations for proportion screened and screening rate among those screened.

#### Observed incidence

The outcome variable used for the model was cumulative incidence comprised of both non-screened and screened cases. We stratified our PLCO sample by birth cohort to get categories of age at study entry and age-specific preclinical incidence rates as described in the previous section. Each risk-factor level (i.e., smokers and nonsmokers) was stratified by birth cohort in order to calculate the age-specific screening rates and proportion screened for each stratum as described in the screening intensity section. To get the overall observed incidence (*G*_*S-R*_*(r)*), the risk-factor-birth-cohort-specific observed incidence was summed across birth cohort strata using as birth-cohort weights, the actual birth-cohort proportions seen in the PLCO population sampled for this study.

A theoretical correction factor can be calculated based on a typical two-by-two table where a is the number of cases with the risk factor, b is the number of noncases with the risk factor, c is the number of cases without the risk factor, and d is the number of noncases without the risk factor. The estimated RR from the simulation is defined here as the ratio between the observed incidence rates in two risk-factor strata (e.g., smoking vs. nonsmoking; *G*_*S-R*_*(r* = 1*)/ G*_*S-R*_*(r* = 2*)*) under the null hypothesis of no association between risk-factor or screening and disease. The unbiased RR between the strata would equal 1 under the null hypothesis; any deviation represents bias. Therefore, to theoretically correct our observed RR (or OR) for any screening bias, we make the expected rates equal (under the null) between the strata by multiplying the incident cases in the denominator stratum (i.e., *c*) by the simulated RR (i.e., expected amount of screening bias). The result is an LTBCA-corrected risk estimate, which assumes that the bias affects all cases proportionately. If this bias varies systematically by some other factor, the model can be further stratified to reflect this. In addition, other bias factors may be applied (e.g., bias from changed incidence due to prevention effects[2])

## Simulation *r*esults

**Fig 6:**
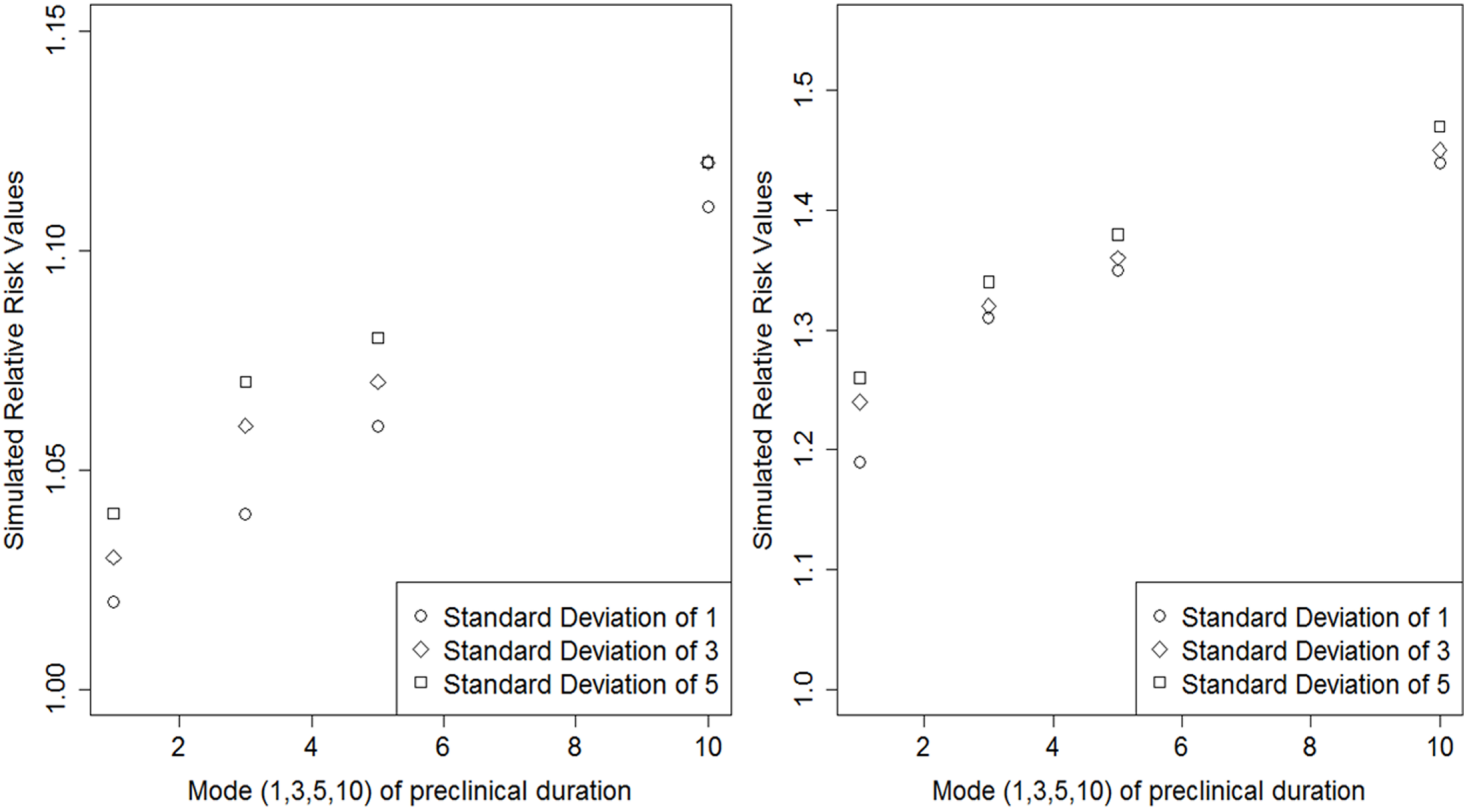
Simulated relative risks for smoking under the double null hypothesis (i.e., smoking and screening are independent of lung cancer) for studies sampling from the entire PLCO enrollment period (1993–2001) in the usual-care group (left; from Table 1a) or from those affected by the procedural modification (1995–2001) in the intervention group (right; from Table 1b). Both studies select cases and sample noncases between study time T3 and study time T5. The 12 relative risks were simulated using a combination of four preclinical duration distribution parameters for the mode (1,3,5,10) and three standard deviations (sd) (1,3,5). The relative risks are comparing the categories “ever smoked” to “never smoked.” The simulation is based on study sample specific age distributions and screening proportion and rates among those screened. Unbiased RR = 1.

The parameter combinations of preclinical duration distributions (mode years of 1, 3, 5, 10; standard deviation years of 1, 3, 5) for the smoked variable categorized into age groups 55–59 and 60–64; 65–69 and 70–74, produced twelve simulated risk ratios for each study (Fig 6). In Fig 6, the graph on the left illustrates that in the study that samples from the entire PLCO enrollment years (93–01) in the usual-care group with a case-ascertainment period of T3-T5 as the mode increases, so does the bias, while the difference in bias between different standard deviation years remains relatively equal. The graph on the right illustrates that in the study sampled from those affected by the procedural modification in the intervention group with case-ascertainment T3-T5 as the mode year increases, so does the bias, while the difference in bias between different standard deviation years decreases. The result from the study done in the usual-care group follows a mostly linear pattern and the study done in with the intervention group follows more of a log pattern.

The tables illustrate that three different chest x-ray sensitivities of 46%, 66%, and 86% were used along with twelve different combinations of the mode years (1,3,5,10) and standard deviation years (1,3,5) for a lognormal preclinical duration distribution. These combinations were repeated for each screening test sensitivity under the assumption the population had a 20% overdiagnosis rate. The overdiagnosis rate is incorporated into the simulation by modifying the representative lognormal preclinical duration distribution for the target population so that 20% of the population are assumed to be drawn from a lognormal preclinical duration distribution with a mode of 20 years and standard deviation of 3 years and the other 80% from one of the previously specified lognormal distributions. So in actuality because the screening exam sensitivity is below 100%, the simulated sample population will not exhibit an overdiagnosis rate of 20% but rather something lower in association with screening sensitivity. In other words, not all 20% of the population with the extremely long preclinical duration will be detected by screening. The association between screening sensitivity and overdiagnosis within a sample population is the probability of being screen detected multiplied by the overdiagnosis rate in the target population.

For the data set sampled from the entire PLCO enrollment period (93–01) in the usual-care group with a case-ascertainment period of T3-T5 and with a chest x-ray screening test sensitivity of 46%, the *RR* range from 1.02 when the mode is 1 and standard deviation is 1 for the lognormal preclinical duration distribution to 1.12 when the mode is 10 and standard deviation is 5 (Table 1a). Adding a 20% overdiagnosis rate the *RR* range is 1.05 to 1.13 (Table 1c). For a screening sensitivity of 66%, the simulated *RR* values range from 1.02 to 1.11 (Table 2a) and with a 20% overdiagnosis rate to these simulations creates the *RR* range of 1.04 to 1.12 (Table 2c). When using a sensitivity of 86%, the simulated *RR* value range changes to 1.01 to 1.10 (Table 3a) and with a 20% overdiagnosis rate the range of *RR* values is 1.03 to 1.11 (Table 3c).

**Table 1:** Simulated *RR*s for the studies sampled from the entire PLCO enrollment period (93–01) in the usual-care group (a and c) and only those affected by the procedural modification (95–01) the intervention group (b and d) using a lognormal distribution for the preclinical duration with modes of 1, 3, 5, and 10 years and standard deviations of 1, 3, 5 years and a constant chest x-ray sensitivity of 46%. To test model sensitivity to overdiagnosis, in the bottom two tables a 20% overdiagnosis rate was applied. Because of imperfect screening sensitivity, the overdiagnosis rate in the simulated sample population is actually less than 20%. Unbiased RR = 1. a) Usual-care group sampled from entire enrollment period: Simulation results using smoked variable: Lognormal distribution for preclinical duration with no overdiagnosis and chest x-ray sensitivity of 46%. b) Intervention group sampled after procedural modification: Simulation results using smoked variable: Lognormal distribution for preclinical duration with no overdiagnosis chest x-ray sensitivity of 46%. c) Usual-care group sampled from entire enrollment period: Simulation results using smoked variable: Lognormal distribution for preclinical duration with 20% overdiagnosis and chest x-ray sensitivity of 46%. d) Intervention group sampled after procedural modification: Simulation results using smoked variable: Lognormal distribution for preclinical duration with 20% overdiagnosis and chest x-ray sensitivity of 46%.

a)	Mode = 1	Mode = 3	Mode = 5	Mode = 10
Standard dev. = 1	1.02	1.04	1.06	1.11
Standard dev. = 3	1.03	1.06	1.07	1.12
Standard dev. = 5	1.04	1.07	1.08	1.12
b)				
Standard dev. = 1	1.19	1.31	1.35	1.44
Standard dev. = 3	1.24	1.32	1.36	1.45
Standard dev. = 5	1.26	1.34	1.38	1.47
c)				
Standard dev. = 1	1.05	1.06	1.08	1.12
Standard dev. = 3	1.06	1.08	1.09	1.13
Standard dev. = 5	1.07	1.08	1.10	1.13
d)				
Standard dev. = 1	1.24	1.35	1.38	1.47
Standard dev. = 3	1.29	1.36	1.40	1.48
Standard dev. = 5	1.31	1.38	1.42	1.50

**Table 2:** Simulated *RR*s for the studies sampled from the entire PLCO enrollment period (93–01) in the usual-care group (a and c) and only those affected by the procedural modification (95–01) the intervention group (b and d) using a lognormal distribution for the preclinical duration with modes of 1, 3, 5, and 10 years and standard deviations of 1, 3, 5 years and a constant chest x-ray sensitivity of 66%. To test model sensitivity to overdiagnosis, in the bottom two tables a 20% overdiagnosis rate was applied. Because of imperfect screening sensitivity, the overdiagnosis rate in the simulated sample population is actually less than 20%. Unbiased RR = 1. a) Usual-care group sampled from entire enrollment period: Simulation results using smoked variable: Lognormal distribution for preclinical duration with no overdiagnosis and chest x-ray sensitivity of 66%. b) Intervention group sampled after procedural modification: Simulation results using smoked variable: Lognormal distribution for preclinical duration with no overdiagnosis and chest x-ray sensitivity of 66%. c) Usual-care group sampled from entire enrollment period: Simulation results using smoked variable: Lognormal distribution for preclinical duration with 20% overdiagnosis and chest x-ray sensitivity of 66%. d) Intervention group sampled after procedural modification: Simulation results using smoked variable: Lognormal distribution for preclinical duration with 20% overdiagnosis and chest x-ray sensitivity of 66%.

a)	Mode = 1	Mode = 3	Mode = 5	Mode = 10
Standard dev. = 1	1.02	1.03	1.05	1.10
Standard dev. = 3	1.03	1.05	1.06	1.10
Standard dev. = 5	1.03	1.05	1.07	1.11
b)				
Standard dev. = 1	1.19	1.31	1.35	1.44
Standard dev. = 3	1.24	1.33	1.37	1.46
Standard dev. = 5	1.27	1.34	1.39	1.48
c)				
Standard dev. = 1	1.04	1.05	1.07	1.11
Standard dev. = 3	1.05	1.06	1.08	1.12
Standard dev. = 5	1.06	1.07	1.09	1.12
d)				
Standard dev. = 1	1.25	1.35	1.39	1.47
Standard dev. = 3	1.29	1.37	1.40	1.49
Standard dev. = 5	1.31	1.38	1.42	1.50

**Table 3:** Simulated *RR*s for the studies sampled from the entire PLCO enrollment period (93–01) in the usual-care group (a and c) and only those affected by the procedural modification (95–01) the intervention group (b and d) using a lognormal distribution for the preclinical duration with modes of 1, 3, 5, and 10 years and standard deviations of 1, 3, 5 years and a constant chest x-ray sensitivity of 46%. To test model sensitivity to overdiagnosis, in the bottom two tables a 20% overdiagnosis rate was applied. Because of imperfect screening sensitivity, the overdiagnosis rate in the simulated sample population is actually less than 20%. Unbiased RR = 1. a) Usual-care group sampled from entire enrollment period: Simulation results using smoked variable: Lognormal distribution for preclinical duration with no overdiagnosis and chest x-ray sensitivity of 86%. b) Intervention group sampled after procedural modification: Simulation results using smoked variable: Lognormal distribution for preclinical duration with no overdiagnosis and chest x-ray sensitivity of 86%. c) Usual-care group sampled from entire enrollment period: Simulation results using smoked variable: Lognormal distribution for preclinical duration with 20% overdiagnosis and chest x-ray sensitivity of 86%. d) Intervention group sampled after procedural modification: Simulation results using smoked variable: Lognormal distribution for preclinical duration with 20% overdiagnosis and chest x-ray sensitivity of 86%.

a)	Mode = 1	Mode = 3	Mode = 5	Mode = 10
Standard dev. = 1	1.01	1.03	1.04	1.08
Standard dev. = 3	1.02	1.04	1.05	1.09
Standard dev. = 5	1.03	1.05	1.06	1.10
b)				
Standard dev. = 1	1.19	1.31	1.35	1.44
Standard dev. = 3	1.24	1.33	1.37	1.46
Standard dev. = 5	1.27	1.34	1.39	1.48
c)				
Standard dev. = 1	1.03	1.05	1.06	1.10
Standard dev. = 3	1.04	1.06	1.07	1.10
Standard dev. = 5	1.05	1.06	1.08	1.11
d)				
Standard dev. = 1	1.25	1.35	1.39	1.47
Standard dev. = 3	1.29	1.37	1.41	1.49
Standard dev. = 5	1.31	1.38	1.42	1.50

For the data set sampling those affected by the procedural modification (95–01) from the intervention group with a case-ascertainment period of T3-T5 and with a chest x-ray screening test sensitivity of 46%, the *RR* range from 1.19 when the mode is 1 and standard deviation is 1 for the lognormal preclinical duration distribution to 1.47 when the mode is 10 and standard deviation is 5 (Table 1b). Adding a 20% overdiagnosis rate, the *RR* range is 1.24 to 1.50 (Table 1d). For a screening sensitivity of 66%, the simulated *RR* values range from 1.19 to 1.48 (Table 2b) and with a 20% overdiagnosis rate to these simulations creates the *RR* range of 1.25 to 1.50 (Table 2d). When using a sensitivity of 86%, the simulated *RR* value range changes to 1.19 to 1.48 (Table 3b) and with a 20% overdiagnosis rate the range of *RR* values is 1.25 to 1.50 (Table 3d).

## Discussion

When a screening test is used among the population in which participants are selected from for an observational study, the screen-detected cases will have an earlier date of diagnosis and likely slower progressing disease compared to non-screen-detected cases. If differential screening behavior exists between risk-factor stratum, case-ascertainment may be changed differentially, and thereby misrepresenting the observed RR between the risk factor of interest and disease. In the presence of differential screening under plausible assumptions about preclinical incidence and duration, the simulations presented here show the possibility for LTBCA because of chest x-ray screening to significantly affect the estimated risk smoking has on the development of lung cancer.

Using two case-control study designs nested within the PLCO randomized trial as simulation examples, a possible range for such bias within the smoking-lung cancer observed risk estimate has been given. Within these results, a relationship has emerged that as screening differential (either in proportion or rate) between strata of the variable (e.g., ever smoked vs. never smoked) increases, so does the susceptibility to this screening bias. Fig 6 illustrates that in general when the mode and standard deviation increase, so does the amount of bias expected to affect the observed RR. Also, the model appears to be slightly sensitive to standard deviation and much more so to mode variations causing simulation values to differ by about 25% when comparing smallest to largest pairs of these parameters (Tables 1–3). This result can be explained by considering that a disease that has a long preclinical duration in combination with an increase in screening use during the study will have the potential to shift many cases into the study that would otherwise not be identified as such. There is an indication in Tables 1c & 1d–3c & 3d that overdiagnosis can have an effect on the RR, most of all when the preclinical duration is shortest (e.g., mode = 1, standard deviation = 1). This observation fits with the previous result that a longer preclinical duration (added by overdiagnosis to the short preclinical duration group) for a disease increases the expected bias affecting the observed RR. The model is only moderately sensitive to screening test sensitivity (average difference is about 2%) comparing simulated values with equal parameters (Tables 1–3) when there are small screening behavior differences (i.e., selecting from the usual-care group). The model is about equally sensitivity to different screening test sensitivity variations when the screening behavior difference between smokers and never smokers is increased (e.g., in the intervention group study).

Because chest x-ray screening for lung cancer has been around for a relatively long time, the estimation of preclinical incidence using the clinical incidence numbers is likely to include screen-detected cases. The impact to bias estimates would occur in the case where proportionally more screen-detected lung cancers are identified at one end of the case-ascertainment period compared to the other (e.g., if screening guidelines/practices change during the ascertainment period). Also, as the baseline (unscreened) incidence of disease increases, the potential for LTBCA would also increase. These points should be considered when interpreting these model results.

The conditions of the case-control studies nested in the PLCO trial (i.e., short study duration) and screening for lung cancer with chest x-ray (i.e., potential for overdiagnosis) are such that considerable bias can arise. Because of chest x-ray screening in this population, the cases observed in the ascertainment period are a different subgroup than the cases expected in the absence of screening. Thus, even if screening is theoretically accounted for with the simulated value, study results should be generalized with caution.

## Supporting information

S1 File Annotated Rcode for producing output. (R)

S2 File Breakdown of model formulae. (DOC)
