# Supplementary material for: A mathematical model of case-ascertainment bias: Applied to case-control studies nested within a randomized screening trial
Source: PLoS One. 2018 Mar 19;13(3):e0194608. doi: 10.1371/journal.pone.0194608 (PMC5858824; doi:10.1371/journal.pone.0194608)
Supplement: S2 File — (DOC) [file pone.0194608.s002.doc]

S2 File

In the model development section, the incidence in the absence of screening (G_U-R_(·)) specific to risk factor (r) and age (a) is given as:

$$G_{U-R}(a_{E,i};r)=\int_{0}^{a_{0,i}} w\_b(x)\int_{a_{0,i}}^{a_{E,i}} f(z-x) dzdx+\int_{a_{0,i}}^{a_{E,i}} w\_d(x)\int_{a_{0,i}}^{a_{E,i}} f(z-x) dzdx$$

This model is identifying the proportion of the population that develop lung cancer before the study (*a_0_*) and becomes symptomatic during the study (*a_0_* to *a_E_*) added to the proportion that develop lung cancer during the study and become symptomatic during the study. To get the cumulative incidence rate for a specific risk-factor stratum (r), we need to sum over all age categories using the age structure of the sampled population (ω) as weights:

*G_U-R_* (*r*) =$\sum_{i} \omega_{i}$ $G_{U-R}(a_{i};r)$

In this mathematical formula as well as the ones that follow, *w*(*x*) represents a quadratic function for preclinical incidence based on the SEER estimated incidence of lung cancer in the absence of screening where *w*(*x*)_b represents the incidence before the case ascertainment period and *w*(*x*)_d represents the incidence after the case ascertainment period. *f*(·) represents the preclinical duration of lung cancer.

The average probability of a subject being screen-detected before the beginning of the study (*a_0_*) given a constant screening test sensitivity (ξ) and age-variable proportion screened (*k_b_1_(·)*) and screening rate (*k_b_2_(·)*) for each risk-factor stratum is given by the following equation:

$$k\_b_{1,r}(x)\left[1-(1-\xi)^{\int_{screenage}^{a_{0,i}} k\_b_{2,r}(y)dy}\right]$$

where *screenage* is the earliest age for screening in the general population (e.g., age 50 for chest X-ray screening for lung cancer). Thus, the complete equation to identify the incidence within each risk-factor stratum that would be moved out of the study (cases detected by screening before the case ascertainment period) that would normally be symptomatically detected during the study is:

$$B_{u,m}(t)=\sum_{m} \omega_{m}\int_{0}^{\max(t,t_{0})} w(x)\int_{\max(x,t_{0})}^{t_{E}} f(z-x)\cdot k_{3}(t)\cdot[1-(1-\xi)^{\int_{\max(screenage,x)}^{t0} k_{1}(y)dy}]dzdx.$$

The incidence in each risk-factor stratum from cases that would have been diagnosed symptomatically after the end of the study period, but are screen-detected during the case-ascertainment period is derived analogously from two different groups. The first group become detectable before t_0_ , but escape detection until after t_0_; the second become detectable after t_0_.

The corresponding average probability of a subject being screen-detected during the study is given by the following fromulae:

$k\_d_{1,r}(x)\left[1-(1-\xi)^{\int_{screenage}^{a_{E,i}} k\_d_{2,r}(y)dy}\right]$ and

$$k\_b_{1,r}(x)(1-\xi)^{\int_{\min(a_{0,i},screenage...)}^{a_{0,i}} k\_b_{2,r}(y)dy}[k\_d_{1,r}(x)\left[1-(1-\xi)^{\int_{a_{0,i}}^{a_{E,i}} k\_d_{2,r}(y)dy}\right]$$

With the combined equations given as:

$$\begin{matrix} A_{u,m}(t)=\int_{0}^{a_{0,i}} w\_b(x)\left[k\_b_{1,r}(x)(1-\xi)^{\int_{\min(a_{0,i},screenage...)}^{a_{0,i}} k\_b_{2,r}(y)dy}[k\_d_{1,r}(x)\left[1-(1-\xi)^{\int_{a_{0,i}}^{a_{E,i}} k\_d_{2,r}(y)dy}\right]\right]\int_{a_{E,i}}^{\max age} f(z-x) dzdx \\ +\int_{a_{0,i}}^{a_{E,i}} w\_d(x)\left[k\_d_{1,r}(x)\left[1-(1-\xi)^{\int_{screenage}^{a_{E,i}} k\_d_{2,r}(y)dy}\right]\right]\int_{a_{E,i}}^{\max age} f(z-x)dzdx \end{matrix}$$

The complete incidence change expected within the study population under screening within each stratum (r) is attained by subtracting the incidence excluded before the case-ascertainment period (B_u,m_) from the incidence included during the case-ascertainment period (A_u,m_).

$$G_{S-R}(a_{E,i};r)=A_{u,m}-B_{u,m}$$

The adjusted cumulative incidence rate for age stratum *i*, *G_S-R_*(*r*), is summed over all age strata *i = 1,…, I*, applying the age structure of the sampled population, *ω_i_*, as weights (same as when we assume population is unscreened).

G_S-R_(r) =$\sum_{i} \omega_{i}$ $G_{S-R}(a_{i};r)$

The ratio of these two cumulative incidences (G_S-R_(r=1)/ G_S-R_(r=2)) is 1 for an unbiased study. Any deviation from 1 represents LTBCA.
